# Madura’s foot: reasons for the delay in diagnosis and consequences for the management (a case report)

**DOI:** 10.11604/pamj.2020.37.75.24983

**Published:** 2020-09-18

**Authors:** Baudouin Boanimbek, Youness Aznague, Guedi Omar Abass, Fath El Khir, Mohamed Amine Benhima, Imad Abkari, Halim Saidi

**Affiliations:** 1 Department of Orthopedic Surgery and Traumatology, Mohammed VI University Hospital, Marrakesh, Morocco

**Keywords:** Madura foot, mycetoma, delayed diagnosis, surgery

## Abstract

Madura foot, relatively easy to diagnose in tropical countries, is very rare and unrecognized in Morocco, causing diagnostic delays. We present the case of a 54-year-old patient with mycetoma for 3 years who initially consulted two general practitioners, then an endocrinologist and finally a dermatologist in order to be diagnosed correctly. The diagnosis of mycetoma based on biological criteria was established at a late stage of irreversible bone lesions; requiring amputation by the orthopedic team. Mycetomas are fungal or bacterial. Delays in diagnosis and care are frequent in Morocco. The diagnosis is based on biology; however, radiological examinations are necessary to assess the extension. The initial treatment is medicinal. Surgery takes place in late stages.

## Introduction

Madura foot or mycetoma is initially a skin condition caused by pathogens of fungal (eumycetoma) or bacterial (actinomycetoma) origin. The mode of transmission is by traumatic inoculation through injured skin, especially at the foot. It is a frequent affection in tropical zones, very rare in our context. The delayed diagnosis, frequent in our context due to the ignorance of this pathology, leads to treatment, by the orthopedic surgery team, at a late stage with destruction of the soft tissues and bone damage.

## Patient and observation

**Figure 1:**
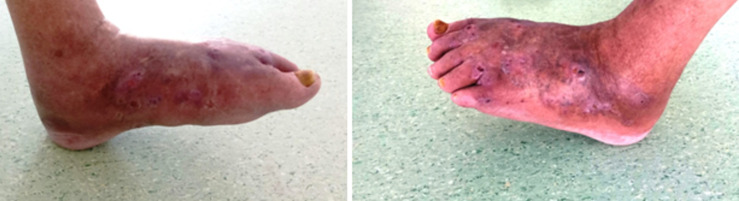
appearance of the patient's foot; we notice the papules and fistulas scattered on the dorsal side of the patient's foot

**Figure 2:**
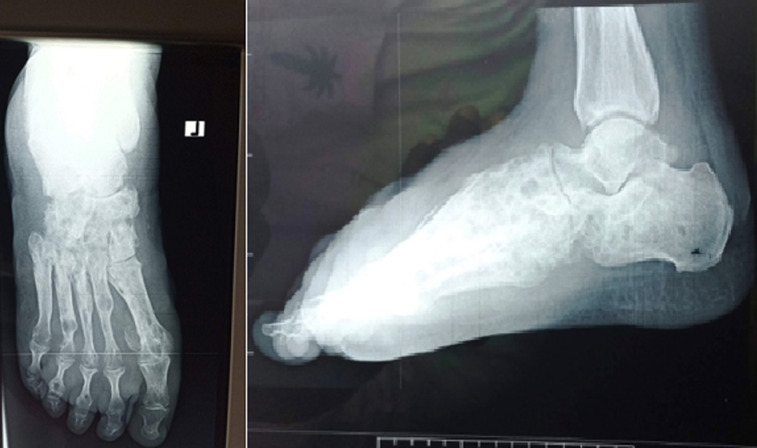
anterior-posterior x-ray of the left foot showing non-specific bone destruction

We report the case of a 54-year-old patient, a farmer living in a rural area, type 2 diabetic on insulin who presented 3 years ago an inflammatory vesicular lesion of the left foot, progressing gradually. The patient consulted a general practitioner 3 months after the onset of the first symptoms, who concluded with contact eczema and put the patient on dermocorticoids. The evolution of the pathology was made by the appearance of fistulas on the dorsal side of the foot. The patient again consulted with a general practitioner who after a disturbed glycemic assessment concluded with a diabetic foot, prescribed a diagram of glycemic balance and antibiotic treatment and care for the foot of the patient. After further treatment failure the treatment was referred to an endocrinologist for diabetic foot. After glycemic control, a dermatological opinion was requested by the endocrinologist in front of the atypical aspect of the foot (Figure 1). New exams were prescribed, including a biopsy with pathology study and an x-ray of the foot (Figure 2). The pathology study revealed the presence of *Madurella mycetomi*. Given the severity of the bone damage, the patient was referred to the orthopedic surgery team for treatment. The decision for a trans-tibial amputation has been made; given the patient's refusal, twice-daily care was offered to him; unfortunately, the patient was lost to follow-up in particular because of the COVID-19 crisis.

## Discussion

Mycetomas are chronic infections of the skin and subcutaneous tissue due to mycotic or bacterial origin. Bone damages are possible in the chronic forms making the severity of the infection [1]. The rarity of the infection under certain skies and the ignorance of the pathology by health professionals often lead to a delay in treatment which can be fatal for the limb [2]. The atypical course of our patient in the various health professionals for 3 years and the erroneous treatments prescribed, are in fact the usual path of the rare cases that we have encountered in our practice. Mycetomas are generally found in tropical regions and can be seen at all ages [3, 4]. The foot is the most common location due to frequent trauma, especially in rural populations walking barefoot, but the infection can occur anywhere in the body [5]. Initially, the mycetoma may present as a papule, a non-mobile skin nodule, a vesicle with a hard base, or a subcutaneous abscess that ruptures to form a fistula on the surface of the skin. Fibrosis is commonly seen in and around early lesions. The pain is minimal or nonexistent except in the case of acute suppurative bacterial infection [6]. The infection progresses slowly for months or years, and there is a progressive extension with destruction of the muscles, tendons, fascia and bones. There is no systemic spread or general symptomatology [7].

In advanced infections, the extremities of the limbs involved appear abnormally swollen, forming a hard, multi-lobed mass, formed of cystic areas. The multiple interconnecting drainage channels and fistulas in these areas secrete thick or serohematic exudates containing characteristic grains, which may be white or black [6]. Biological diagnosis is an essential step to confirm the diagnosis; several stages are necessary: collection and direct examination of the grains in the event that they are emitted, culture of the grains, biopsy and anatomopathological examination [6]. Bone damage must also be systematically sought via a radiological assessment; standard x-rays can show non-specific bone destruction, ultrasound can show suggestive images [8]. The initial treatment of mycetomas is medicated, by the administration of antibiotics or antifungals depending on whether the causative agent is bacterial or fungal. However, the delay in diagnosis and management in advanced stages requires surgical management. The choice between debridement, localized excision and amputation depends on the extent of the lesion, soft tissue infiltration and bone damage [9]. Unfortunately in our context, given the stage of advanced bone destruction in which we receive patients, radical surgical treatment is usually undertaken. Transtibial amputation is then performed and subsequent fitting allows social and professional reintegration of patients.

## Conclusion

Madura foot or mycetoma is in our context a serious pathology systematically putting into play the functional prognosis of the limb, which is most often amputated because of delayed diagnosis and management. Relatively easy to diagnose in tropical areas, it remains an exception in our context, hence the need for prevention and training of health personnel working in rural areas.
